# Tracheal stenting for primary tracheal mucosa-associated lymphoid tissue lymphoma

**DOI:** 10.1186/2047-783X-18-8

**Published:** 2013-04-02

**Authors:** Juanjuan Ding, Zhuochang Chen, Manli Shi

**Affiliations:** 1 Respiratory Department, HeNan Provincial People’s Hospital, No 7 Weiwu Road, Zhengzhou 450003, China; 2 Pathology Department, HeNan Provincial People’s Hospital, No 7 Weiwu Road, Zhengzhou 450003, China

**Keywords:** Lymphoma, Mucosa-associated lymphoid tissue (MALT), Trachea, Tracheal stent

## Abstract

Primary tracheal mucosa-associated lymphoid tissue (MALT) lymphoma is extremely rare. We report a 49-year-old female patient with the complaint of dyspnea. Fiberoptic bronchoscopy showed polypoid, variable-sized and irregular nodules causing narrowing of the tracheal lumen from the proximal trachea to the left main bronchus. Because of severe stenosis in the airway and the severity of symptoms, this case was unresectable. The patient was then treated successfully with placement of an endobronchial stent through bronchofibroscopy. After the placement of the stent, bronchoscopic biopsy was performed. Pathological analysis confirms a diagnosis of MALT-associated malignant lymphoma. We performed systemic chemotherapy on the patient. The temporary stent was removed after the reduction of the stenosis. This is the first case in which tracheal MALT lymphoma was treated successfully following tracheal stent insertion guided by bronchofibroscopy. Temporary tracheal stenting can be a favorable choice for a patient with tracheal stenosis caused by primary tracheal MALT lymphoma.

## Background

Mucosa-associated lymphoid tissue (MALT) lymphoma is a distinct subgroup of non-Hodgkin’s lymphoma (NHL) that comprises more than two-thirds of all primary NHL of the lung [1]; however, primary tracheal MALT lymphoma is quite rare. Thus, there is no guideline for the treatment of the disease. Surgery, chemotherapy, and radiation therapy usually are selected according to the clinical stage. This is the first report of primary tracheal MALT lymphoma in China that was successfully treated with temporary tracheal stent insertion guided by bronchoscopy and systemic chemotherapy. A review of the literature regarding primary MALT lymphoma is provided to better define the clinical characteristics and management of this uncommon disease.

## Case presentation

**Figure 1:**
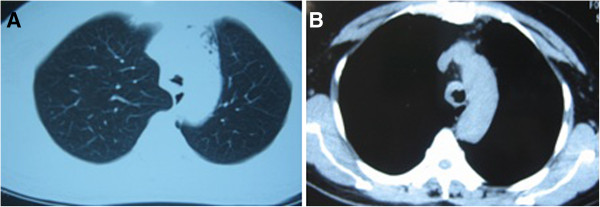
**Computed tomography (CT) scan of the chest showed multiple soft tissue masses adjacent to the trachea, causing diffuse narrowing of the tracheal lumen.** (**A**) pulmonary window. (**B**) mediastinal window.

**Figure 2:**
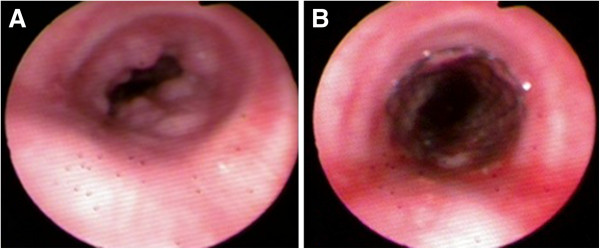
**The presentation of fibrobronchoscopy showed polypoid, variable-sized and irregular nodules.** Narrowing of the tracheal lumen from the proximal trachea to the left main bronchus (**A**). The patency of the lumen after stent implantation (**B**).

**Figure 3:**
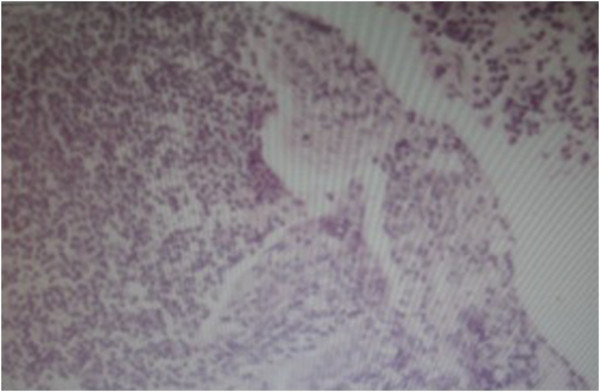
Microscopic appearance of tracheal tissue (hematoxylin and eosin stain, 200x) showing proliferation of lymphoid cells beneath the intact tracheal mucosa.

**Figure 4:**
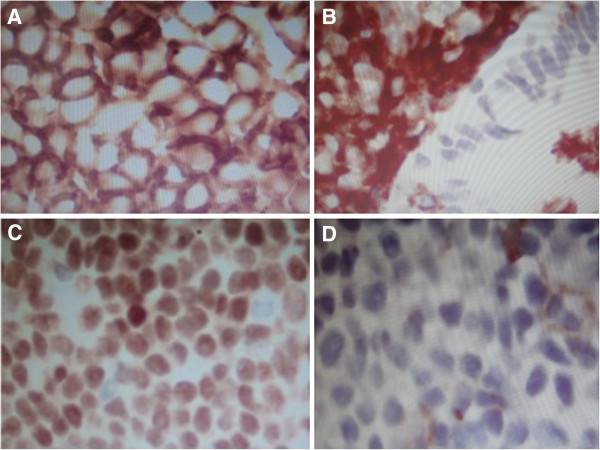
**The immunohistochemical results (DAB, 400x) showed the presence of markers.** LCA (**A**), CD20 (**B**), Pax-5 (**C**) and CD43 (**D**).

A 49-year-old woman was referred to our hospital with the complaint of dyspnea for 4 years and increasing symptoms over the last 2 months. She did not present with night sweats or fever. Her medical, smoking and family histories were all noncontributory. Inspiratory and expiratory coarse rhonchi were auscultated over the lung. Her other physical examination was unrevealing. A computed tomography (CT) scan of the chest showed soft tissue masses adjacent to the trachea and causing diffuse narrowing of the tracheal lumen; however, there was no evidence of mediastinal lymphadenopathy (Figure 1). Fiberoptic bronchoscopy showed polypoid, variable-sized and irregular nodules causing narrowing of the tracheal lumen from the proximal trachea to the left main bronchus (Figure 2A). Because of the severe stenosis of the airway, the body of the fiberoptic bronchoscope failed to pass the lumen of the trachea. The risk when using an Nd:YAG laser and high frequency electrocautery via bronchofibroscopy is high. Thus, we decided to place the endobronchial stent in the trachea (Figure 2B). After the successful placement of stent, bronchoscopic biopsy was performed. The histological finding indicated a dense proliferation of lymphoid cells beneath the intact tracheal mucosa. The shape and size of these lymphoid cells are almost identical (Figure 3). The immunohistochemical results showed the presence of markers LCA, CD20, Pax-5 and CD43, and the absence of CD5 and CD23 (Figure 4). Pathological analysis confirms a diagnosis of MALT-associated malignant lymphoma. For the staging of the primary disease, we performed a contrast-enhanced CT scan of the abdomen and chest. There was no evidence of lymphoma involvement. The patient received a bone marrow biopsy, a gastric fibroscopy, and a color Doppler ultrasound of the neck. These examinations revealed no abnormalities. Next, the patient was given six courses of CHOP chemotherapy (cyclophosphamide 750 mg/m^2^ day 1, adriamycin 50 mg/m^2^ day 1, vincristine 1.4 mg/m^2^ day 1, and prednisone 100 mg/day 1 to 5, q 3 weeks). The tracheal stent was extracted after 35 days after the patient had been given one course of chemotherapy. Currently, the patient has been well without any symptoms of recurrence for 12 months since the initial diagnosis.

## Discussion

In 1984, Isaacson and Wright [2] described extra-nodal lymphomas arising from MALT. Although MALT lymphoma occurs most frequently in the gastrointestinal tract, they can also rise in various non-gastrointestinal sites, such as the salivary gland, conjunctiva, thyroid, orbit, lung, breast, kidney, skin, liver, uterus, and prostate [3]. Among the non-gastrointestinal MALT lymphomas, the pulmonary lymphomas are the most frequent, with figures of up to 19% among MALT lymphomas [4]. In spite of this, a primary presentation of MALT lymphoma affecting the trachea is very rare. Most reports in the literature are case reports.

Clinical features of MALT-derived lymphomas include a tendency to remain localized for prolonged periods, and, thus, to be responsive to locally directed therapy. Owing to the rarity of the primary tracheal MALT lymphoma, standard treatment protocols have not yet been optimized. Local treatment such as surgical resection, radiotherapy and intervention therapy by bronchofibroscopy are generally efficacious, and comprehensive treatment is preferred. We present the case of a female patient with primary tracheal MALT lymphoma who was treated successfully with the placement of a temporary endobronchial stent through the use of fiberoptic bronchoscopy, followed by systemic chemotherapy.

Typically, in these cases, fiberoptic bronchoscopy shows intratracheal polypoid lesions with the tracheal lumen narrowing in one lumen. However our patient presented with the lesions localized from the trachea to the left main bronchus. This case has characteristics in common with the case reported by Kang *et al.*[5], whose case showed multiple, variable-sized nodules causing bronchial narrowing from the proximal trachea to bronchi of both lungs, including the carina. The lesions of both of these cases are systemic disseminated.

To relieve airway obstruction and provide opportunities for bronchoscopic biopsy in our case, we placed a self-expanding metal stent via flexible bronchoscopy in the trachea. To our knowledge, this is the first case of stent placement in primary tracheal MALT lymphoma. Temporary airway stenting has been extensively performed to palliate dyspnea caused by extrinsic compression, intraluminal disease, and loss of cartilaginous support. It is not a cure, but is used mainly to help patients with various diseases breathe better. Bernd *et al.*[6] investigated this strategy of temporary airway stenting in five consecutive patients with malignant lymphoma who presented with severe dyspnea. The results show that temporary stenting is a valuable strategy in chemo- and radiosensitive malignancies, as it ameliorates the patient’s respiratory condition until tumor-specific therapy is effective. Numerous studies have reported that endobronchial therapies, including airway stenting, can palliate symptoms in 80 to 97% of patients with dyspnea [7]. Patients with significant central airway obstruction are frequently within hours or days of dying due to suffocation [8]. Airway resection and reconstruction provides the most reliable definitive correction, but many patients are unresectable. For these patients therapeutic bronchoscopy provides rapid palliation that can be lifesaving and improve quality of life. In general, temporary tracheal stenting as palliation treatment can be a favorable choice for a patient with tracheal stenosis by primary tracheal MALT lymphoma. Also, it is an integral part of multi-therapy methods.

## Conclusions

In conclusion, primary tracheal MALT lymphoma is seen rarely, but it responds well to therapies such as surgery, chemotherapy, and radiation therapy, used either alone or in combination. Temporary tracheal stenting followed by chemotherapy and/or radiotherapy is beneficial in the management of tracheal lymphoma with symptomatic airway stenosis, as in this case.

## Consent

Written informed consent was obtained from the patient for publication of this case report and any accompanying images. A copy of the written consent is available for review by the Editor-in-Chief of this journal. This study is retrospective, and it is approved by the ethics board.

## Abbreviations

NHL: non-Hodgkin’s lymphoma; MALT: Mucosa-associated lymphoid tissue.

## Competing interests

The authors declare that they have no competing interests.

## Authors’ contributions

DJJ was involved in data acquisition, analysis and interpretation, and in drafting the manuscript. ZCC was involved in data analysis and in the critical revision of the manuscript. MLS participated in the revision of the manuscript. All authors read and gave final approval for the version submitted for publication.

## References

[B1] Arnaoutakis K Oo TH Bronchus-associated lymphoid tissue lymphomas South Med J 2009 102 1229 1233 10.1097/SMJ.0b013e3181bfdd2d 20016430

[B2] Isaacson P Wright DH Extranodal malignant lymphoma arising from mucosa-associated lymphoid tissue Cancer 1984 53 2515 2524 10.1002/1097-0142(19840601)53:11<2515::AID-CNCR2820531125>3.0.CO;2-C 6424928

[B3] Zinzani PL Poletti V Zompatori MB Tani M Spaggiari L Tomassetti S Broccoli A Derenzini E Baccarani M Bronchus-associated lymphoid tissue lymphoma: an update of a rare extranodal maltoma Clin Lymphoma Myeloma 2007 7 566 572 10.3816/CLM.2007.n.042 18186964

[B4] Malek SN Hatfield AJ Flinn IW MALT Lymphomas Curr Treat Options Oncol 2003 4 269 279 10.1007/s11864-003-0002-2 12943607

[B5] Kang JY Park HJ Lee KY Lee SY Kim SJ Park SH Kim YK Extranodal marginal zone lymphoma occurring along the trachea and central airway Yonsei Med J 2008 49 860 863 10.3349/ymj.2008.49.5.860 18972610 PMC2615368

[B6] Schmidt B Massenkeil G John M Arnold R Witt C Temporary tracheobronchial stenting in malignant lymphoma Ann Thorac Surg 1999 67 1448 1450 10.1016/S0003-4975(99)00254-4 10355429

[B7] Lund ME Garland R Ernst A Airway stenting: applications and practice management considerations Chest 2007 131 579 587 10.1378/chest.06-0766 17296664

[B8] Wood DE Liu YH Vallières E Karmy-Jones R Mulligan MS Airway stenting for malignant and benign tracheobronchial stenosis Ann Thorac Surg 2003 76 167 172 10.1016/S0003-4975(03)00033-X 12842534

